# Co-administration with DNA encoding papillomavirus capsid proteins enhances the antitumor effects generated by therapeutic HPV DNA vaccination

**DOI:** 10.1186/s13578-015-0025-y

**Published:** 2015-06-25

**Authors:** Benjamin Yang, Andrew Yang, Shiwen Peng, Xiaowu Pang, Richard B.S. Roden, T.-C. Wu, Chien-Fu Hung

**Affiliations:** Departments of Pathology, School of Medicine, Johns Hopkins University, CRBII Room 307, 1550 Orleans Street, Baltimore, MD 21231 USA; Obstetrics and Gynecology, Johns Hopkins Medical Institutions, Baltimore, MD USA; Molecular Microbiology and Immunology, Johns Hopkins Medical Institutions, Baltimore, MD USA; Oncology, Johns Hopkins Medical Institutions, Baltimore, MD USA; Department of Oral Pathology, College of Dentistry, Howard University, Washington, DC USA

**Keywords:** Capsid, DNA vaccine, Human papillomavirus (HPV)

## Abstract

**Background:**

DNA vaccines have emerged as attractive candidates for the control of human papillomavirus (HPV)-associated malignancies. However, DNA vaccines suffer from limited immunogenicity and thus strategies to enhance DNA vaccine potency are needed. We have previously demonstrated that for DNA vaccines encoding HPV-16 E7 antigen (CRT/E7) linkage with calreticulin (CRT) linked enhances both the E7-specific CD8^+^ T cell immune responses and antitumor effects against E7-expressing tumors. In the current study, we aim to introduce an approach to elicit potent CD4^+^ T cell help for the enhancement of antigen-specific CD8^+^ T cell immune responses generated by CRT/E7 DNA vaccination by using co-administration of a DNA vector expressing papillomavirus major and minor capsid antigens, L1 and L2.

**Result:**

We showed that co-administration of vectors containing codon-optimized bovine papillomavirus type 1 (BPV-1) L1 and L2 in combination with DNA vaccines could elicit enhanced antigen-specific CD8^+^ in both CRT/E7 and ovalbumin (OVA) antigenic systems. We also demonstrated that co-administration of vectors expressing BPV-1 L1 and/or L2 DNA with CRT/E7 DNA led to the generation of L1/L2-specific CD4^+^ T cell immune responses and L1-specific neutralizing antibodies. Furthermore, we showed that co-administration with DNA encoding BPV1 L1 significantly enhances the therapeutic antitumor effects generated by CRT/E7 DNA vaccination. In addition, the observed enhancement of CD8^+^ T cell immune responses by DNA encoding L1 and L2 was also found to extend to HPV-16 L1/L2 system.

**Conclusion:**

Our strategy elicits both potent neutralizing antibody and therapeutic responses and may potentially be extended to other antigenic systems beyond papillomavirus for the control of infection and/or cancer.

## Background

The knowledge that high risk human papillomavirus (HPV) is a necessary etiological factor for the development of cervical cancer provides the opportunity for control of cervical cancer and/or other HPV-associated malignancies through vaccination against HPV. Currently, the two commercially available prophylactic vaccines, Gardasil and Cervarix, based upon virus-like particles (VLP) derived from the major capsid protein L1 effectively protect against infection by the two most common HPV types found in cervical cancer (for review see [1]). The minor capsid antigen L2 also shows promise for preventive vaccination in animal models, although it is less immunogenic than L1 VLP. However, neither L1 VLP nor L2-based vaccines generate therapeutic effects against established HPV infection [2]. Therefore, given the significant burden of HPV-associated lesions worldwide, there is an urgent need to develop therapeutic HPV vaccines for the control of existing HPV infection and associated malignancies. Quite different from preventive vaccines which target the viral capsid proteins L1 and/or L2, therapeutic HPV vaccines focus on targeting the HPV E6 and E7, since only these oncoproteins are consistently expressed in HPV-associated cancers and are responsible for the malignant transformation.

Among the various therapeutic HPV vaccines currently being tested, DNA vaccines have emerged as attractive candidates for the treatment of cervical cancer and associated malignancies. Naked DNA is relatively safe, stable, and easy to produce and transport (for review see [3]). Furthermore, DNA vaccines are capable of sustained cellular gene expression, promote MHC class I antigen presentation, and have the capacity for repeated administration since they do not lead to the generation of neutralizing antibodies. However, an important limitation of DNA vaccines is limited potency since they lack the intrinsic ability to amplify and spread *in vivo*. Therefore, it is important to consider strategies to improve DNA vaccine potency strategies (for review, see [4, 5]).

One strategy to enhance DNA vaccine potency is to improve antigen expression, processing, and presentation in antigen-presenting cells using intracellular targeting strategies (for review, see [4, 5]). Several of our previous studies have employed DNA vaccines encoding calreticulin (CRT) linked to HPV-16 E7 antigen (CRT/E7) [6–11] because this fusion greatly enhances the E7-specific CD8^+^ T cell immune responses in vaccinated mice. This phenomenon likely reflects the efficient recognition of calreticulin by dendritic cells and improved targeting to the MHCI pathway by targeting to the endoplasmic reticulum [6].

Activation and proliferation of CD4^+^ T cells is crucial to the success of both humoral and cell-mediated responses to viral infection. Although the fusion of E7 with CRT enables such DNA vaccines to elicit significant E7-specific CD8 T cell immunity in the absence of CD4 T cell help [12], approaches to boost CD4^+^ T cell help are likely to enhance DNA vaccine potency. Indeed, CD4^+^ T cells play a significant role in priming effector CD8^+^ T cells, thus augmenting the CD8^+^ T cell responses, as well as generation of memory T cell populations (for review see [13]). CD4^+^ T cells help to differentiate naïve CD8^+^ T cells into effector cells by providing activation signals to dendritic cells (DCs), most notably IL-2, thus promoting CD8^+^ T cell proliferation. Thus, strategies to induce CD4^+^ T helper cells at sites of CD8^+^ T cell priming can potentially enhance CTL immune responses.

In the current study, we aim to combine intracellular targeting strategies using CRT with a strategy to enhance CD4^+^ T help for the development of a therapeutic HPV DNA vaccine. Since papillomavirus L1 or L2 antigens likely contain CD4^+^ T cell epitopes [14–18], we reasoned that co-administration of vectors containing codon-optimized bovine papillomavirus (BPV) L1 and L2 may provide CD4^+^ T cell help and enhance the antigen-specific CD8^+^ T cell immune responses generated by DNA vaccines. An additional potential benefit would be the induction of neutralizing antibody and protective immunity [19, 20]. We showed that co-administration of vectors containing BPV L1 or L2 DNA in combination with DNA vaccines could elicit enhanced antigen-specific CD8^+^ in both CRT/E7 and ovalbumin (OVA) antigenic systems. We also demonstrated that co-administration of vectors containing BPV1 L1 ± L2 DNA with CRT/E7 DNA led to the generation of L1/L2-specific CD4^+^ T cell immune responses as well as L1-specific neutralizing antibodies. Furthermore, we showed that co-administration with BPV1 L1 significantly enhances the therapeutic antitumor effects generated by CRT/E7 DNA vaccination. In addition, the observed enhancement of CD8^+^ T cell immune responses by DNA encoding L1 and L2 was also found to extend to HPV-16 L1/L2 system. Overall, our data suggest that co-administration of DNA encoding papillomavirus L1 or L2 can be used to enhance antigen-specific CD8^+^ T cell immune responses generated by therapeutic HPV DNA vaccination for the control of HPV infection and HPV-associated tumors. Furthermore, our approach can also generate neutralizing antibodies against papillomavirus for potential prevention against infection. This strategy also provides the opportunity to combine preventive and therapeutic approaches. Our strategy may potentially be extended to other antigenic systems for the control of infection and/or cancer.

## Results

### Co-administration with vectors encoding papillomavirus L1 or L2 significantly enhances the antigen-specific CD8^+^ T cell immune responses generated by CRT/E7 or OVA DNA vaccination

**Fig. 1:**
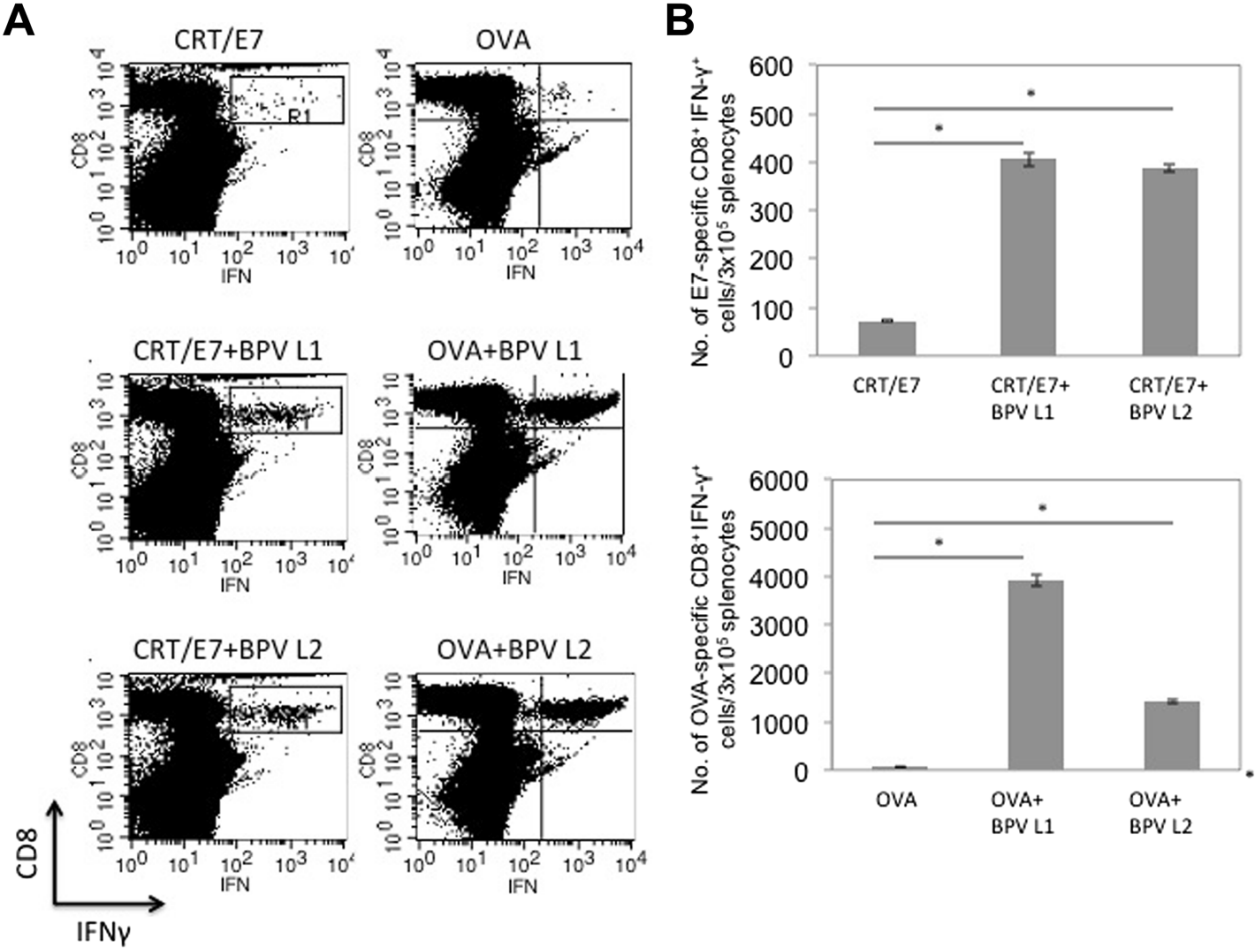
Characterization of antigen-specific CD8^+^ T cell immune responses generated by antigen-specific DNA vaccine mixed with vectors containing BPV1 L1 or L2 DNA. C57BL/6 mice (five per group) were vaccinated intradermally via gene gun with 2 μg/mouse of CRT/E7 or OVA DNA with or without BPV1 L1 or L2 DNA twice at 1-week intervals. Splenocytes from vaccinated mice were collected 1 week after last immunization and the E7 or OVA-specific T cell immune responses were characterized using intracellular cytokine staining followed by flow cytometry analysis. **a** Representative flow cytometry data depicting the number of E7 (upper panel) or OVA(lower panel)-specific CD8^+^ T cells. **b** Bar graph representing the number of E7 (upper panel) or OVA (lower panel) -specific CD8^+^ T cells/3x10^5^ splenocytes (mean ± SD). Data shown are representative of two experiments performed. * indicates *p* < 0.05

**Fig. 2:**
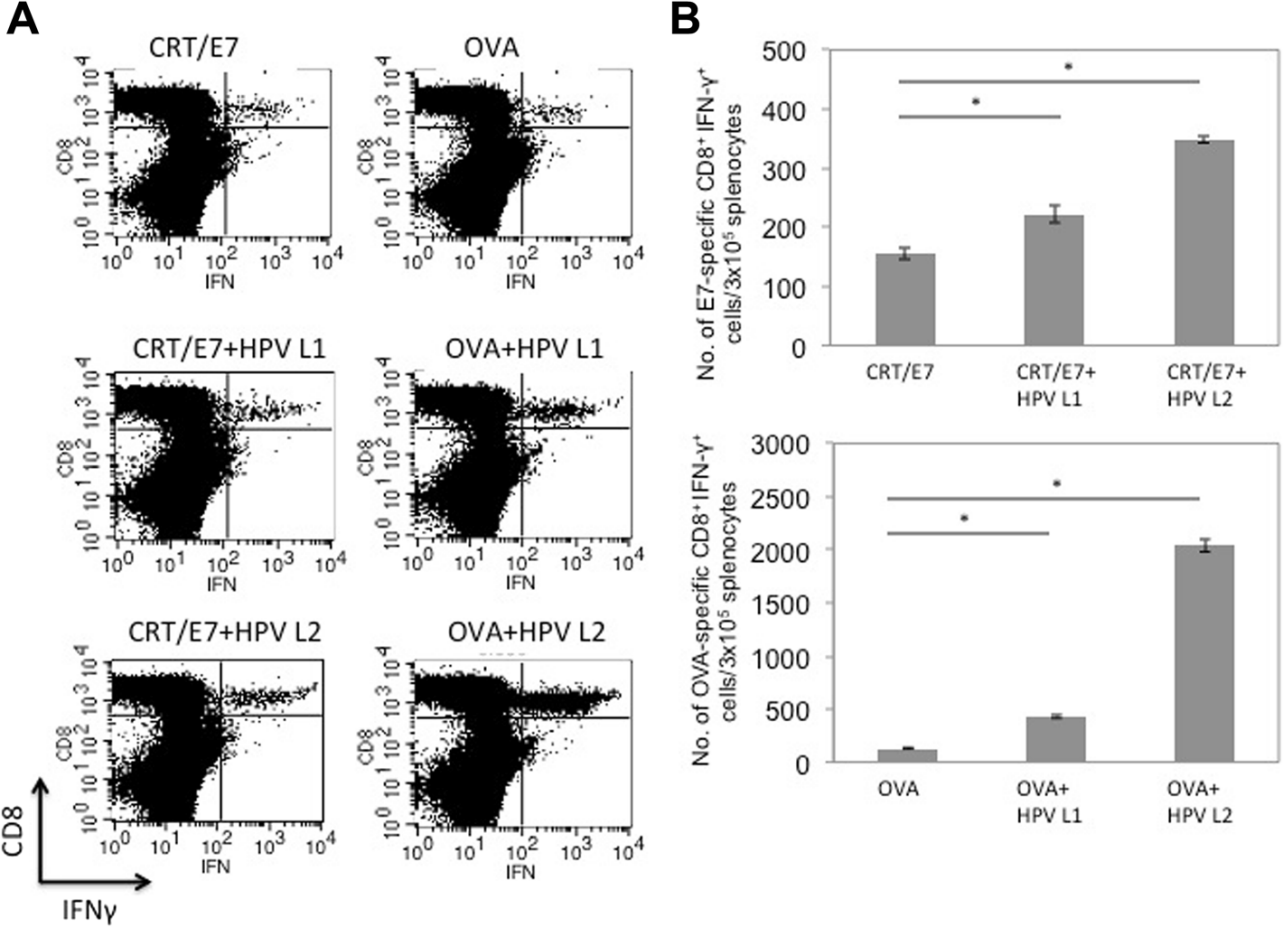
Characterization of antigen-specific CD8^+^ T cell immune responses generated by antigen-specific DNA vaccine mixed with HPV-16 L1 or L2 DNA. C57BL/6 mice (five per group) were vaccinated intradermally via gene gun with 2 μg/mouse of CRT/E7 or OVA DNA with or without HPV-16 L1 or L2 DNA twice at 1-week intervals. Splenocytes from vaccinated mice were collected 1 week after last immunization and the E7 or OVA-specific T cell immune responses were characterized using intracellular cytokine staining followed by flow cytometry analysis. **a** Representative flow cytometry data depicting the number of E7 (upper panel) or OVA (lower panel)-specific CD8^+^ T cells. **b** Bar graph representing the number of E7 (upper panel) or OVA (lower panel)-specific CD8^+^ T cells/3x10^5^ splenocytes (mean^±^ SD). Data shown are representative of two experiments performed. * indicates *p* < 0.05

In order to characterize the antigen-specific CD8^+^ T cell immune responses generated by vaccination with CRT/E7 or OVA DNA in combination with vectors containing codon-optimized BPV1 L1 or L2 DNA, C57BL/6 mice (five per group) were vaccinated intradermally via gene gun with CRT/E7 or OVA DNA with or without BPV1 L1 or L2 DNA twice at 1-week intervals. Splenocytes from vaccinated mice were collected 1 week after last immunization and the E7 or OVA-specific T cell immune responses were characterized using intracellular cytokine staining followed by flow cytometry analysis. As shown in Fig. 1, mice vaccinated with CRT/E7 or OVA DNA vaccine in combination with BPV1-L1 or L2 DNA generated significantly higher E7-specific and OVA-specific CD8^+^ T cell immune responses compared to mice vaccinated with CRT/E7 or OVA DNA alone. To evaluate whether the enhancement of antigen-specific CD8+ T cell immune responses by co-administration of BPV1 L1 or L2 DNA is also observed in other papillomavirus systems, we co-administered HPV16 L1 or L2 DNA with CRT/E7 or OVA DNA vaccination. Of note, co-administration of HPV16 L1 or L2 DNA with CRT/E7 or OVA DNA vaccination also generated significantly higher E7-specific CD8+ T cell responses compared to CRT/E7 or OVA DNA vaccination alone in mice (Fig. 2). Thus, our data indicate that co-administration with papillomavirus L1 or L2 DNA significantly enhances the antigen-specific CD8^+^ T cell immune responses generated by DNA vaccination.

### Co-administration of papillomavirus L1 or L2 DNA with CRT/E7 or OVA DNA led to the generation of L1/L2-specific CD4^+^ T cell immune responses

**Fig. 3:**
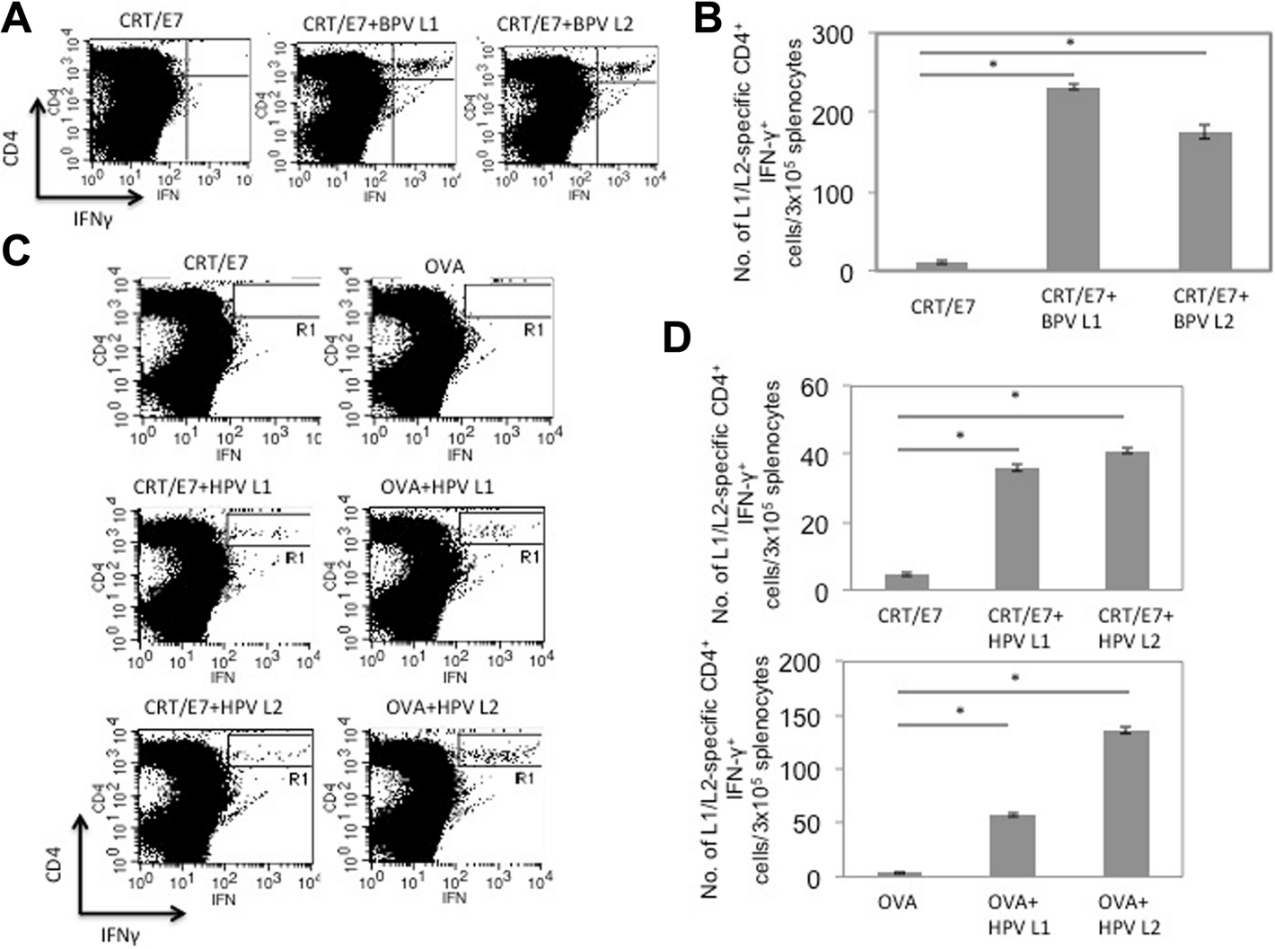
Characterization of BPV1 and HPV-16 L1 or L2-specific CD4^+^ T cell immune responses generated by CRT/E7 or OVA DNA mixed with DNA encoding BPV1 or HPV-16 L1 or L2. C57BL/6 mice (five per group) were vaccinated intradermally via gene gun with 2 μg/mouse of CRT/E7 with or without BPV1 or HPV-16 L1 or L2 DNA twice at 1-week intervals. Splenocytes from vaccinated mice were collected 1 week after last immunization and pulsed with 5 μg/mL of BPV1 or HPV-16 L1/L2 VLPs. The BPV1 L1/ L2-specific CD4^+^ T cell immune responses were characterized using intracellular cytokine staining followed by flow cytometry analysis. **a** Representative flow cytometry data depicting the number of BPV1 L1/L2-specific CD4^+^ T cells. **b** Bar graph representing the number of BPV1 L1/L2-specific CD4^+^ T cells/3x10^5^ splenocytes (mean^±^ SD). **c** Representative flow cytometry data depicting the number of HPV-16 L1/L2-specific CD4^+^ T cells. **d** Bar graph representing the number of HPV-16 L1/L2-specific CD4^+^ T cells/3x10^5^ splenocytes (mean^±^ SD). Data shown are representative of two experiments performed. * indicates *p* < 0.05

In order to determine whether the co-administration with BPV1 L1 or L2 DNA with CRT/E7 DNA will lead to the generation of L1 or L2-specific CD4^+^ T cell immune responses, C57BL/6 mice (five per group) were vaccinated intradermally via gene gun with CRT/E7 DNA with BPV1 L1 or L2 DNA. Mice vaccinated with CRT/E7 DNA alone were used as negative controls. Splenocytes from vaccinated mice were collected 1 week after last immunization and incubated with BPV1 L1/L2 virus-like particles (VLPs). The L1 or L2-specific CD4^+^ T cell immune responses were characterized using intracellular cytokine staining followed by flow cytometry analysis. As shown in Fig. 3a and b, mice vaccinated with BPV1 L1 in combination with CRT/E7 DNA led to the generation of L1-specific CD4^+^ T cell immune responses. Similarly, vaccination with BPV1 L2 with CRT/E7 DNA led to significant level of L2-specific CD4^+^ T cell immune responses compared to vaccination with CRT/E7 alone. Again, to observe whether the CD4+ T cell responses elicited by this vaccination strategy extend to other papillomavirus systems, we co-administered HPV16 L1 or L2 DNA with CRT/E7 or OVA DNA vaccination. As shown in Fig. 3c and d, compared to vaccination with CRT/E7 or OVA DNA alone, mice vaccinated with HPV-16 L1 or L2 DNA in combination with CRT/E7 or OVA DNA led to the generation of L1 or L2-specific CD4^+^ T cell immune responses. These results suggest that co-administration with DNA encoding papillomavirus L1 or L2 with CRT/E7 DNA was able to generate appreciable L1 or L2-specific CD4^+^ T cell immune responses respectively in vaccinated mice.

### Co-administration with BPV1 L1 DNA significantly enhances the therapeutic antitumor effects generated by CRT/E7 DNA vaccination

**Fig. 4:**
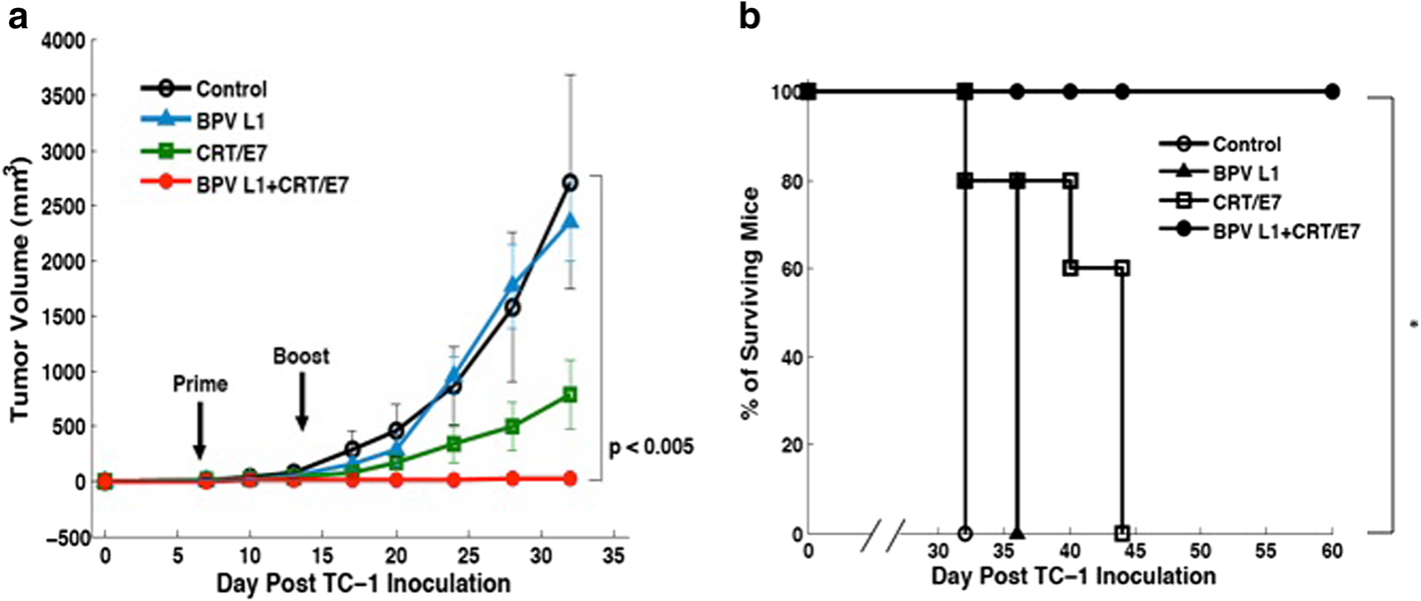
*In vivo* tumor treatment experiments in mice vaccinated with CRT/E7 DNA mixed with BPV1 L1 DNA. C57BL/6 mice (five per group) were first challenged with 1x10^5^/mouse of TC-1 tumor cells subcutaneously. One week after tumor challenge, mice were treated intradermally via gene gun with 2 μg/mouse of CRT/E7 DNA alone, BPV1 L1 DNA alone or CRT/E7 DNA in combination with BPV1 L1 DNA. Vaccinated mice were boosted twice at 1-week intervals with the same dose and regimen. Mice were sacrificed on day 30 after the last vaccination. Tumor growth were monitored twice weekly by caliper measurements and palpations. **a** Line graph depicting the tumor volume over time of tumor-bearing mice treated with CRT/E7 DNA and/or BPV1 L1 DNA. **b** Kaplan-Meier survival analysis of tumor-bearing mice treated with CRT/E7 DNA and/or BPV1 L1 DNA. The data presented are from one representative experiment of the two performed. * indicates *p* < 0.05

In order to determine if the observed enhancement of antigen-specific CD8^+^ T cell immune responses by co-administration of BPV1 L1 DNA can translate into potent therapeutic antitumor effects, we performed in vivo tumor treatment experiments using an HPV-16 E7-expressing murine tumor cell line, TC-1. TC-1 also expresses HPV16 E6, but does not contain either L1 or L2. C57BL/6 mice (five per group) were first challenged with TC-1 tumor cells subcutaneously. One week after tumor challenge, mice were treated intradermally via gene gun with CRT/E7 DNA alone, BPV1 L1 DNA alone or CRT/E7 DNA in combination with BPV1 L1 DNA. Vaccinated mice were boosted twice at 1-week intervals with the same dose and regimen. Tumor growth were monitored twice weekly by caliper measurements and palpations. As shown in Fig. 4, tumor-bearing mice treated with CRT/E7 DNA vaccine in combination with BPV1 L1 DNA generated significantly reduced tumor volume and prolonged survival compared to mice treated with CRT/E7 DNA alone or BPV1 L1 DNA alone. Thus, our data indicate that co-administration with BPV1 L1 DNA is capable of significantly enhancing the therapeutic antitumor effects generated by CRT/E7 DNA vaccination.

### The enhancement in E7-specific CD8+ T cell immune responses are contributed by CD4+ T helper cells

In order to determine the mechanism underlying the observed enhancement of antigen-specific CD8+ T cell immune responses generated by coadministration with L1 or L2 DNA vectors, we have generated pcDNA3 encoding the reverse sequences of L1 (BPV L1 (−)) or L2 DNA (BPV L2 (−)). C57BL/6 mice (five per group) were vaccinated intradermally via gene gun with CRT/E7 DNA with or without the reverse sequence of BPV1 L1 or L2 DNA twice at 1-week intervals. Splenocytes from vaccinated mice were collected 1 week after last immunization and the E7-specific T cell immune responses were characterized using intracellular cytokine staining followed by flow cytometry analysis. We found that mice vaccinated with CRT/E7 DNA vaccine in combination with the reverse sequence BPV1-L1 or L2 DNA did not lead to the increased frequency of E7-specific CD8^+^ T cell immune responses observed in mice vaccinated with CRT/E7 DNA with BPV1-L1 or L2 DNA (data not shown). The insert sequences does not express into L1 and L2 that activate CD4+ T cell help, thereby cannot enhance the E7-specific CD8+ T cell responses of the CRT/E7 vaccine. The result suggests that CD4+ T cell help generated by co-expression of L1 or L2 protein promotes E7-specific CD8+ T cell immune responses to CRT/E7 DNA vaccination.

### Co-administration of BPV1 L1 DNA with CRT/E7 DNA led to the generation of L1-specific neutralizing antibodies

**Fig. 5:**
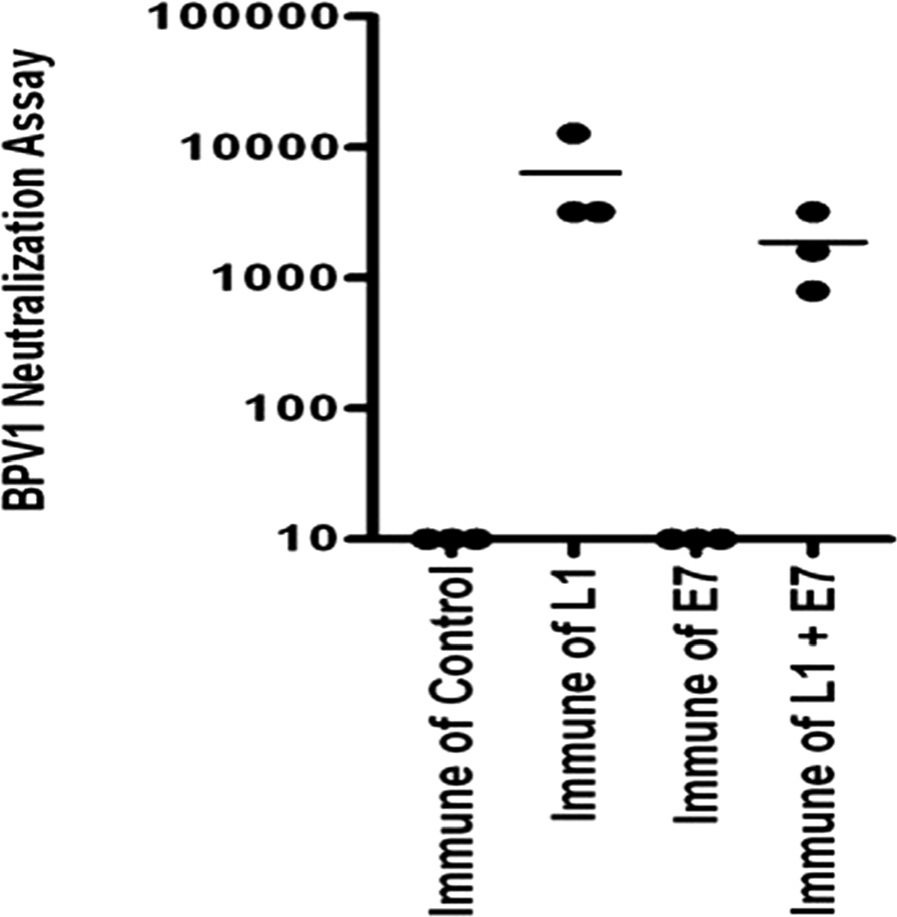
Characterization of BPV1-specific neutralizing antibody responses generated by mice vaccinated with CRT/E7 and/or BPV1 L1 DNA. C57BL/6 mice (three per group) were immunized on days 1, 15, and 30 intradermally using a gene gun with 2 μg of DNA per mouse of CRT/E7 and/or BPV1 L1 DNA. In vitro neutralization assays were performed using BPV1 L1 pseudovirus on twofold dilutions of antisera collected from the mice 2 weeks after the final immunization. Endpoint titers achieving 50 % neutralization are plotted and the means shown as horizontal lines. PI = Pre Immune; L1 = BPV1 L1 DNA vaccine; E7 = CRT/E7 DNA vaccine

In order to determine if co-administration of BPV1 L1 DNA with CRT/E7 DNA will lead to the generation of BPV1 L1-specific neutralizing antibodies, C57BL/6 mice (three per group) were immunized on days 1, 15, and 30 intradermally via gene gun with CRT/E7 and/or BPV1 L1 DNA. In vitro neutralization assays were performed using BPV1 L1 pseudovirus on twofold dilutions of antisera collected from the mice 2 weeks after the final immunization. Mice vaccinated with CRT/E7 in combination with BPV1 L1 DNA were found to generate similar neutralizing antibody responses compared to mice vaccinated with BPV1 L1 DNA alone (Fig. 5).

### Co-administration of BPV1 L1 or L2 DNA with OVA DNA vaccination generated OVA-specific CD8+ T cell response through intramuscular administration

**Fig. 6:**
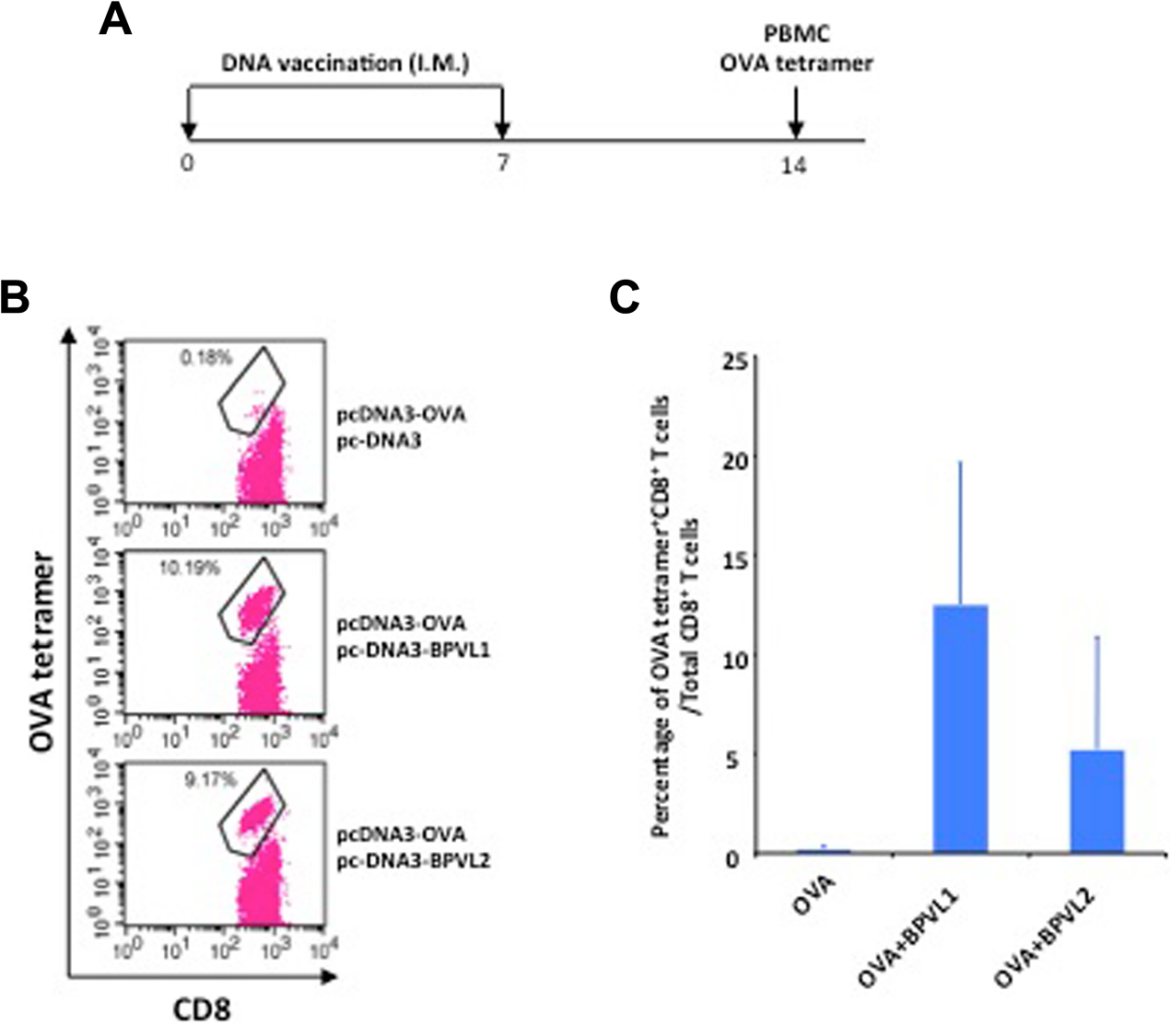
Comparison of OVA-specific CD8^+^ T cell responses induced by pcDNA3-OVA vaccination with or without co-administration of BPV L1 or L2. **a**. Schematic illustration of the experiment. Briefly, 5 ~ 8 weeks old female C57BL/6 mice (3 mice/group) were vaccinated with 10 μg/mouse of pcDNA3-OVA, with either 10 μg/mouse of pcDNA3, or pcDNA3-BPVL1, or pcDNA3-BPVL2 via intramuscular injection. The mice were boosted with the same regimen once after one week. 7 days after the last vaccination, PBMCs were collected from peripheral blood, stained with FITC-conjugated anti-mouse CD8a antibody, PE-conjugated OVA peptide (SIINFEKL) loaded H-2K^b^ tetramer. The data were acquired with FACSCalibur and analyzed with CellQuest. **b**. Representative flow cytometry image of PBMC staining. **c**. Summary of the flow cytometry data

Finally, we evaluate whether co-administration with papillomavirus L1 or L2 DNA can elicit potent antigen-specific CD8+ T cell responses when applied through different route of administration. C57BL/6 mice (three per group) were vaccinated intramuscularly with OVA DNA with or without BPV1 L1 or L2 DNA twice at one-week intervals. One week after the last immunization, PBMCs were collected and the OVA-specific CD8+ T cell immune responses were characterized through flow cytometry analysis. As shown in Fig. 6, mice vaccinated intramuscularly with OVA DNA in combination with BPV1 L1 or L2 DNA generated significantly higher percentage of OVA-specific CD8+ T cells compared to mice vaccinated intramuscularly with OVA DNA alone. This data indicates that co-administration with papillomavirus L1 or L2 DNA can lead to enhanced antigen-specific CD8+ T cell immune responses through the intramuscular route of administration.

## Discussion

In the current study, we showed that co-administration of vectors containing codon-optimized BPV1 or HPV-16 L1 or L2 in combination with DNA vaccines could elicit enhanced antigen-specific CD8^+^ in both CRT/E7 and ovalbumin (OVA) antigenic systems. We also demonstrated that co-administration of BPV1 or HPV-16 L1 or L2 DNA with CRT/E7 DNA led to the generation of L1/L2-specific CD4^+^ T cell immune responses. In addition, the observed enhancement of E7-specific CD8^+^ T cell immune responses by L1 DNA also confers improved therapeutic antitumor effects against an E7-expressing tumor. Moreover, co-administration of BPV1 L1 DNA induced generation of L1-specific neutralizing antibodies, which may serve to prevent further papillomavirus infections. Of note, the enhanced antigen-specific CD8+ T cell responses are observed in intradermal as well as intramuscular routes of administration. Indeed, we have already shown that vaccination with vector expressing L1 is protective against vaginal challenge with papillomavirus pseudovirions [20]. Taken together, our study suggests the promise of this approach for future clinical translation, and it can potentially be applied to other antigenic systems.

Here we show that CD4+ T cell help plays an important role in the enhancement of antigen-specific CD8+ T cell immune responses observed in our vaccination regimen as co-administration with the reverse sequence of BPV1 L1 or L2 did not enhance the antigen-specific CD8+ T cell responses. Studies have shown that L1 and L2 are generally much more effective in eliciting CD4+ T cell responses [14–18]. While it is possible that BPV L1 or L2 could elicit some CD8+ T cell responses, we believe the CD4+ T cell help generated by L1 or L2 DNA is the main contributor to the enhanced antigen-specific CD8+ T cell response observed. Furthermore, it has been shown that CD4+ T cells can help generate memory T cells [21, 22]. Though the current study focuses on characterizing the antigen-specific CD8+ T cell therapeutic antitumor effect by our vaccination strategy, it will be of interest for future studies to further characterize the complete effect of CD4+ T cell and its ability to generate memory T cell responses for prolonged protection.

Our vaccination strategy was able to generate a potent therapeutic antitumor effect in tumor-bearing mice. Interestingly, co-administration of BPV1 L1 DNA with CRT/E7 DNA generated L1-specific neutralizing antibodies, which confers prophylactic value. It has also been shown that vaccination with L1 DNA induced L1-specific neutralizing antibodies in Balb/c mice [20]. Therefore, our vaccination strategy of co-administration of L1 DNA can generate potent antibody responses in more than one genetic background. Of note, studies have shown that papillomavirus L2 is generally not as effective in generating L2-specific neutralizing antibodies [20, 23], thus co-administration with L1 DNA should be prioritized in future translation.

In the current study we show that co-administration of BPV1 L1 DNA with CRT/E7 led to potent therapeutic antitumor effects and prolonged survival due to the enhanced antigen-specific CD8+ T cell responses by CD4+ T cell help. Since co-administration with HPV16 L1 DNA with CRT/E7 also significantly enhances antigen-specific CD8+ T cell responses by CD4+ T cell help, we believe that potent therapeutic antitumor effects should also be observed. To further promote clinical translation, subsequent investigations focusing on directly characterizing the antitumor effects of co-administrating HPV16 L1 with CRT/E7, and whether reversing the sequence of HPV16 L1 abolishes the antigen-specific CD8+ T cell response enhancement should be conducted. In addition, since our vaccination regimen achieved complete tumor suppression, the frequency of vaccination may be modified and further studied to determine the optimal vaccination regimen. Importantly, our data show that the current vaccination strategy can generate enhanced antigen-specific CD8+ T cell responses by both intradermal and intramuscular vaccination. Since DNA vaccines are commonly applied intramuscularly in the clinic, future translation of the current vaccination technology may focus on the intramuscular route of administration.

Several strategies have been employed to induce CD4^+^ T helper cells to enhance antigen-specific CD8^+^ T cell-mediated immune responses generated by therapeutic HPV DNA vaccines. We have previously demonstrated that a DNA vaccine encoding invariant chain (Ii) with the class II-associated invariant peptide (CLIP) region replaced with the pan HLA-DR binding epitope (PADRE) could elicit potent PADRE-specific CD4^+^ T cell responses in vaccinated mice [24]. In addition, a co-administration of this DNA construct (Ii-PADRE) with DNA encoding HPV-16 E7 generated significantly greater CD8^+^ T cell immune responses relative to a co-administration of DNA encoding HPV-16 E7 with DNA encoding unmodified Ii [24]. Thus, the current study represents another promising approach to enhance CD4^+^ T help for the improvement of DNA vaccine potency, but with the added benefit of inducing prophylactic immunity too. It will be of interest in the future to perform a head-to-head comparison between vectors containing BPV L1/L2 DNA with Ii-PADRE DNA for their ability to enhance antigen-specific CD8^+^ T cell responses by therapeutic HPV DNA vaccines. Such information will facilitate the selection of the most effective or desirable DNA construct for improving DNA vaccine potency.

Another potential mechanism for enhancing the therapeutic HPV DNA vaccine potency through the co-administration with L1/L2 DNA is the potential formation of VLPs in transfected cells. It has been shown that the expression of L1 can lead to the formation of VLPs in eukaryotic cells [25]. Furthermore, previous studies have demonstrated that papillomavirus VLPs can directly activate dendritic cells and thereby increase expression of costimulatory markers and MHC class I and II molecules [26–28]. Thus, the co-administration of L1 DNA may lead to the local activation of DCs, resulting in further enhancement of antigen-specific CD8^+^ T cell immune responses generated by DNA vaccination. However, since the L2 only expression construct had a similar effect to L1 DNA, this suggests that the presence of VLP does not explain the enhanced CD8 T cell response upon co-administration with CRT-E7 or OVA constructs.

Strategies to enhance CD4^+^ T cell help may potentially be combined with other strategies to further enhance DNA vaccine potency. We have previously demonstrated a significant enhancement of DNA vaccine potency by combining a strategy to prolong dendritic cell life and intracellular targeting strategies with a strategy to boost CD4^+^ T cell help [29]. Since all these strategies function via different mechanisms, the combination may potentially result in significantly enhanced antigen-specific immune responses and improved antitumor effects. For clinical translation, it will be desirable to identify the best combination of the different strategies in order to achieve the best DNA vaccine potency.

## Conclusions

In summary, our study demonstrates that the employment of DNA encoding papillomavirus L1 or L2 can lead to generation of antigen-specific CD4^+^ T cells and neutralizing antibodies, resulting in the improvement of therapeutic and preventive HPV DNA vaccine potency. Our strategy may potentially be extended to other antigenic systems for the control of infection and/or cancer.

## Materials & Methods

### Mice

C57BL/6 mice (6–8 weeks old) were purchased from the National Cancer Institute (Frederick, MD). All animals were maintained under specific pathogen-free conditions at the Johns Hopkins Hospital (Baltimore, MD). All procedures were performed according to the Johns Hopkins Institutional Care and Use Committee approved protocols and in accordance with recommendations for the proper care of laboratory animals.

### Cells and DNA constructs

TC-1 cells were obtained by co-transformation of primary C57BL/6 mouse lung epithelial cells with HPV-16 E6 and E7 and an activated ras oncogene as described previously [30]. They were maintained in RPMI medium supplemented with 2 mM glutamine, 1 mM sodium pyruvate, 20 mM 4-(2-hydroxyethyl)-1-piperazineetha-nesulfonic acid (HEPES), 5x10^5^ M β-mercaptoethanol, 100 IU ml^−1^ penicillin, 100 μg ml^−1^ streptomycin, 10 % fetal bovine serum, and cultured at 37 °C in a humidified incubator with 5 % CO_2_. The pcDNA3-OVA and pcDNA3-CRT/E7 DNA constructs were generated as described previously [6, 31].

### DNA vaccination

DNA-coated gold particles were prepared as described previously [32]. DNA-coated gold particles were delivered to the shaved abdominal region of mice using a helium-driven gene gun (Bio-Rad Laboratories Inc., Hercules, CA, USA) with a discharge pressure of 400 p.s.i. C57BL/6 mice were immunized with 2 μg of plasmid DNA to each mouse encoding pcDNA3-CRT/E7 mixed with pcDNA3-BPV1-L1 or L2 or HPV-L1 or L2 delivered to the shaved abdomen. The mice received a homologous boost 1 week later.

### C57BL/6 mice were vaccinated with 10 μg of pcDNA3-OVA DNA with 10 μg of pcDNA3, pcDNA3-BPVL1, or pcDNA3-BPVL2 intramuscularly in the thigh muscle. The mice received a homologous boost 1 week later

#### Intracellular cytokine staining and flow cytometry analysis

Splenocytes were harvested from mice 1 week after the last vaccination. Prior to intracellular cytokine staining, 5x10^6^ splenocytes from each vaccination group were incubated for 16 h with 1 μg ml^−1^ HPV-16 E7 H-2Db epitope (RAHYNIVTF) or OVA peptide (ISQAVHAAHAEINEAGR) [31], or 5 μg/mL BPV1 or HPV-16 L1/L2 VLPs in the presence of GolgiPlug (BD Pharmingen) (1 μl ml^−1^). The stimulated splenocytes were then washed once with FACScan buffer and stained with phycoerythrin- conjugated monoclonal rat anti-mouse CD8α or CD4. Cells were subjected to intracellular cytokine staining using the Cytofix/Cytoperm kit according to the manufacturer’s instructions (BD Pharmingen). Intracellular IFN-γ was stained with fluorescein isothiocyanate-conjugated rat anti-mouse IFN-γ to identify the immune response and cytokine levels. PBMCs were collected from peripheral blood 1 week after last intramuscular vaccination and stained with anti-mouse CD8α and OVA peptide (SIINFEKL) loaded H-2K^b^ tetramer. Flow cytometry analysis was performed using FACSCalibur with CELLQuest software (BD Biosciences, Mountain View, CA, USA).

### *In vivo* tumor treatment experiments

C57BL/6 mice (five per group) were inoculated subcutaneously with 1xl0^5^ TC-1 tumor cells per mouse on the left flank. After 1 week, when tumor progression is usually observed, mice were vaccinated with DNA constructs pcDNA3-BPV1 L1 or L2 or pcDNA3-HPV L1 or L2 in conjunction with pCDNA3-CRT/E7 or pcDNA3-OVA or the control empty vector. A homologous boost was administered 1 week after the first immunization. Mice were monitored for tumor growth by measuring diameters with calipers twice a week.

### Neutralization assays

The BPV1 L1, L2 and HPV L1, L2 pseudovirions with encapsulated secreted alkaline phosphatase (SEAP) were generated by co-transfection of 293TT cells with plasmids encoding BPV1 L1, L2 or HPV L1 and L2 and a SEAP reporter plasmid as described previously [33]. Cells collected after transfection were treated overnight with Brij 58 (0.5 %), Benzonase (0.5 %) and purified by centrifugation on an Optiprep step gradient (27, 33, and 39 %) at 40,000 rpm for 4.5 h. Pseudovirus neutralization assays were carried out as outlined previously [34]. Briefly, the pseudovirus and the pooled mouse immune sera were incubated for 1 h and the mixture was used to infect 293TT cells. 68–72 h post-infection, the supernatants were collected and SEAP activity in the supernatants was measured by colorimetric assay. Serum neutralization titers were defined as the highest dilution that caused at least a 50 % reduction in SEAP activity, compared to control pre-immune serum samples.

### Statistical analysis

All data expressed as means ± s.d. are representative of at least two different experiments. Data for intracellular cytokine staining with flow cytometry analysis and tumor treatment experiments were evaluated by analysis of variance. Comparisons between individual data points were made using Student’s *t*-test. All p values < 0.05 were considered significant.

## Abbreviations

HPV: Human papillomavirus
CRT: Calreticulin
BPV: Bovine papillomavirus
OVA: Ovalbumin
DCs: Dendritic cells
VLPs: Virus-like particles
CLIP: Class II-associated invariant peptide
PADRE: Pan HLA-DR binding epitope
SEAP: Encapsulated secreted alkaline phosphatase
